# Caregivers’ care experiences of children with developmental dislocation of the hip in Tibet, China: a convergent mixed-methods study

**DOI:** 10.3389/fped.2025.1561246

**Published:** 2025-05-09

**Authors:** Yang Lei, Jiang Yan, Shao Wenjuan, Shen Weili, Xiao Qianyi, Fan Lingyan

**Affiliations:** Department of Orthopedics, Shanghai Children’s Hospital, School of Medicine, Shanghai Jiao Tong University, Shanghai, China

**Keywords:** developmental dislocation of the hip, care capacity, care burden, social support, convergent mixed-methods

## Abstract

**Aim:**

To understand, comprehensively, care experiences of caregivers for children with developmental dislocation of the hip in Tibet, China.

**Background:**

Postoperative rehabilitation for children with developmental dislocation of the hip (DDH) may last for several months to years. Even after discharge, recovery is often partial. It is important to note that despite optimal surgical treatment, lifelong residual issues may persist, such as limping, chronic pain, or early-onset osteoarthritis. Home care is critical in postoperative recovery. Family as the basic unit of home-based care for patients, many measures are inevitably carried out in the family environment and rely on family caregivers to provide. This requires caregivers to pay long-term and high attention, posing significant challenges to caregiving capacity.

**Design:**

A convergent mixed-methods.

**Methods:**

Caregivers (*n* = 76) completed the Chinese version of the Family Caregiver Care Ability Scale, the Zarit Caregiver Burden Scale and 12 participated in semi-structured interviews from September, 2023 to February 2024. Descriptive statistics and Pearson correlation analysis were used for quantitative analysis, thematic analysis for qualitative analysis. Both quantitative and qualitative data were merged and integrated for mixed-methods analysis.

**Results:**

Low caregiver capacity and moderate caregiver burden scores were reported. Four themes emerged from care experiences: different emotions at different stages (initial worry, backend trust), caregiving process overwhelmed (role conflict, lack of caregiving capacity), insufficient information (disease-specific information, actual care information), and social support.

**Conclusion:**

This study is the first to use this mixed-methods design to analyze the current state of care and burden among family caregivers of children with developmental dislocation of the hip in Chinese highland region-Tibet. The combined results showed that qualitative results converged with quantitative results in terms of emotional changes, role burden, lack of disease-specific information, and social support; qualitative results complemented quantitative results in terms of caregiving capacity deficits and lack of actual care information to explain the quantitative results. The quantitative findings also emphasized the link between social support in terms of caregiving capacity and caregiving burden. These results guide future research to promote rehabilitation and improve quality of life for affected children and families.

## Introduction

Developmental dysplasia of the hip (DDH) represents a spectrum of disorders affecting the development of the hip joint and is one of the most common congenital malformation in pediatric orthopedics (1). Clinically, it mostly occurs in infants and young children, and will gradually develop symptoms such as limitation of hip joint movement and pain during growth (2). The pathogenesis of DDH is not well understood, and it is currently believed that disease progression intertwines genetic susceptibility with external mechanical forces on the developing hip (3). Such forces affect the developmental window *in utero* and shortly after birth and may distort the alignment of femoral head with acetabulum. DDH is influenced by a variety of factors and the etiology involves both genetic and non-genetic factors. Literatures reported that positive family history, gender (often female), breech birth were risk factors contributed to development of this disorder (4). In addition, the “bundled” swaddling position with legs extended, hips extended and adducted can deteriorate the immature hip joint and increase the risk of DDH (5). Maternal hormones (e.g., estrogen) may contribute to development of DDH, but perinatal hormones (e.g., “relaxin”) are more likely to contribute to DDH (6). Some studies have shown the incidence of DDH is related to race, with higher rates in Canda and North America, followed by Africa and China (7). The prevalence of DDH had been reported to be about 1% in worldwide (8) and about 0.09%–0.30% in China (9, 10). This rate varied by region, for example, in Shanghai, a coastal city in eastern China, the prevalence of DDH was nearly 0.91% (11), while in Shigatse-Tibet, a high-altitude city in western China, the prevalence was as high as 32.4% (12). Currently, the occurrence of late primary diagnosis of DDH has been greatly reduced by early neonatal hip ultrasound screening in developed countries and in cities such as Shanghai and Beijing in China. But in western part of China, especially in underdeveloped areas such as Tibet, many children with late primary diagnosis of DDH existed due to weakness of healthcare base. Therefore, growth and development of Tibetan children deserve attention of medical professionals.

DDH involves ligaments, femoral head and joint capsule, resulting in loss of normal relationship between bone and acetabulum in joint capsule, making joint dislocated or subluxated. It includes three types of acetabular dysplasia, subluxation and dislocation. Main manifestations are limping, swaying, limitation of limb movement, etc., which affecting development and growth of children (13). At present, there are still controversies internationally regarding the treatment and duration of DDH in children. The Graf method for ultrasound diagnosis and grading are currently the most used and accepted method for DDH diagnosis and grading (14, 15). This method can be used to guide the selection of intervention methods as well as predict treatment outcomes. Early diagnosis and timely treatment are key points of DDH. At present, surgery combined with plaster fixation was mostly used to achieve purpose of treatment (16). However, there remains a risk of complications, such as avascular necrosis (AVN), which may occur not only after surgery but also folloeing treatment with the Pavlik harness. This complication can impair the growth of the femoral head or acetabulum, potentially compromising long-term outcomes (16, 17). Some researchers found recovery of hip function in children with DDH has a direct impact on efficacy of surgery, and the guarantee of surgical efficacy depends on continuous postoperative care, rehabilitation exercises, daily mobility exercises and prevention of complications (18). Inadequate caring may result in trauma to hip joint and surrounding soft tissues or adhesions within joint due to hematoma, which even lead to serious consequences such as joint re-dislocation, joint stiffness, and so on (19). Therefore, good postoperative care and rehabilitation is quite crucial.

Based on characteristics of disease, most children will return home to carry out rehabilitation training on their own after a period of treatment in hospital. Due to the young age of children, most of their caring and rehabilitation training was done with assistance of family caregivers, which put corresponding demands on caregiving ability of family caregivers. It has been shown that active involvement of family caregivers in caregiving can shorten days of hospitalization and promote recovery (20). However, good home care was more effective in preventing complications, promoting improvement of limb function, daily ability and quality of life. Relevant studies have confirmed that family caregivers of children with DDH are not sufficiently knowledgeable about disease awareness and caregiving skills (20–22). Many caregivers lack various types of information during treatment, such as disease information (etiology, clinical manifestations, etc.); preoperative information (preoperative preparations, surgical procedures, etc.); postoperative information (pain control measures, skin care, dietary management, etc.) and discharge information (time of review, care measures during immobilization in a cast or brace, functional limb exercises, etc.). Furthermore, researchers concluded family caregivers were less capable of providing home care in four areas of living care, plaster care, skin care, and exercise guidance (21). This showed family caregivers need to improve their knowledge and skills in caring for their children.

Scholars have focused on analysis of home care for children with DDH in low altitude areas, there is a lack of research on caregiving experiences of family caregivers of children with DDH in high altitude areas. Therefore, a need exists to further understand family caregivers’ experiences and feelings to evaluate their caring attitude and skills.

## This study

### Aims

To gain an in-depth understanding of the caregiving burden and caregiving experience felt by family caregivers accompanying children with DDH during hospitalization after surgical treatment in the highland region of western China-Tibet, as an indicator of family caregiving management. This study focused on a few specific questions:

1. What are the caregiving burdens perceived by family caregivers when administering care to children with DDH during hospitalization?
2. What is the family caregiver's ability to provide care to the children with DDH during hospitalization?
3. What are the family caregivers’ perceptions of the caregiving experience? To what extent are these perceptions consistent with their experiences?

### Design

A convergent mixed-methods design was used in this study, where the quantitative and qualitative components had equal, and separate, simultaneous status in the study. Data was compared, contrasted, transformed, and correlated when analyzing.

### Setting

This study was conducted in the orthopedic ward of a tertiary pediatric hospital in Shanghai, China. Many children with DDH come to here every year for treatment because this hospital is running a public assistance program, “Love of Gesang Flower”, for children with DDH in Tibet, a high-altitude region in western China. DDH families usually stayed in hospital for 10–14 days until their vital signs were stabilized, then staff arranged for a train to take them back to Tibet. Caregivers lived with child during treatment. The orthopedic ward set up with beds for caregivers to accompany their children, making it easy for caregivers to check on their child's status at any time. All costs for the treatment were covered by that public assistance program at no additional costs.

### Sample

Inclusion criteria included: (a) being the primary caregiver of the child, providing care without compensation; (b) caring for a child diagnosed with DDH by x-ray (unilateral or bilateral) and treatment of hip joint open reduction + pelvic + femoral osteotomy; (c) having good language skills (if the caregiver was an ethnic minority who was unable to speak Mandarin, an interpreter or a translator should be provided) and agreeing to participate in the study. The exclusion criteria included: (a) mental abnormality; (b) those who were unable to fill out the questionnaire completely or answers to entries were consecutively the same; and (c) those who participated in other research projects during the research period.

During the quantitative study, a total of 80 questionnaires were distributed and 80 were recovered, of which 4 had the phenomenon of consecutively identical responses, thus 76 questionnaires were valid, with an overall validity rate of 95%. During the qualitative phase of the study, 12 family caregivers were recruited through convenience sampling. A small qualitative sample size is justified as it provides a deeper understanding of the phenomenon under study (23).

### Data collection

Data collection period was from September 2023 to June 2024. The same researcher was responsible for data collection to ensure consistency. Prior to data collection, participants were provided with complete information about the study and signed a written informed consent form. The researcher stayed with participants during the administration of scale and answered their questions. They were reminded of their right to withdraw. After completing the questionnaire, participants were asked if they were willing to join in a 10–30 minutes semi-structured interview. Interviews were generally scheduled on the 3rd day after surgery. The reasons for conducting the interview were explained to those family members who were willing to be interviewed, and then they were asked to be interviewed in a quiet place. The whole interview process avoided using induced cues, summarized responses of family caregivers at the right time, and recorded the interviewee's true and comprehensive experiences, views, opinions, knowledge and attitudes after obtaining his or her consent. The orthopedic demonstration room was selected as the interview site. The audio recordings were converted into textual form within 24 h. Two researchers collected information, and used thematic analysis to refine themes.

The Chinese version of Family Caregiver Task Inventory (FCTI), revised by Lee and Mok (24) of Hong Kong, was used in this study. It includes 25 items in 5 dimensions: (a) learning to cope with new role; (b) providing care according to care-receiver's needs; (c) managing own emotional needs; (d) appraising supportive resources; and (e) balancing caregiving needs and one's own needs. A 3-point Likert scale was used, with a score of 0 indicating no difficulty, 1 indicating difficulty, and 2 indicating extreme difficulty, for a total score of 0–50, with higher scores meaning poorer caregiving ability. Caregiving burden was measured using the Chinese version of the Zarit Caregiver Burden Interview (ZBI), which was localized by Wang Lie et al (25). It consisted of 22 entries in 2 dimensions: role burden and personal burden. A 5-point Likert scale was used (0 = no, 1 = occasional, 2 = sometimes, 3 = often, 4 = always) and a total score of 0–88, with higher scores indicating heavier caregiving burdens. Social Support Rate Scale (SSRS) was designed by Xiao in 1993 to reflect individual's level of social support (26). Questionnaire has 10 entries, including three dimensions: objective support, subjective support and utilization of social support. The total score ranges from 12 to 66, with higher scores indicating a better level of social support. Lower than 33 scores indicate a low level of social support, 33–45 scores show an average level, and higher than 45 scores indicate a good level of social support.

Qualitative data was collected using a semi-structured interview guide that included the following questions:

• How did you care for your child after she/he underwent surgery, including diet, skin, cast or brace, psychological, daily life, etc.?
• What difficulties or burdens have you faced in caring for your child after she/he received treatment?
• What were some of funny times you had while caring for your child after she/he received treatment?
• Please describe your caregiving abilities specifically.

### Ethical considerations

The study was reviewed by the Ethics Committee of the Shanghai Children's Hospital under approval number 2023R066-E01. We assured that their treatment would not be affected if they refused to participate in this study. No identifying information was collected from the study subjects during research process and the privacy of their beliefs and opinions was ensured. In addition, we encrypted the data so that only researchers could access that.

### Data analysis

SPSS 25.0 was used for statistical analysis. The Shapiro–Wilk test was used to assess the normality of the data, which showed an approximately normal distribution (*P* > 0.05). Total caregiving ability and caregiving burden scores were calculated by recording items under each scale and then summing scores. Qualitative data was analyzed using thematic analysis, including data familiarization and transcription, initial coding, thematic search, thematic review, thematic definition and naming, and thematic finalization (27). Finally, a joint presentation table was developed by integrating quantitative and qualitative information, identifying similar and dissimilar results. Data was combined by comparing the information to select confirming, disconfirming, or expanding results (23).

### Rigor/validity and reliability

To ensure the reliability of interview outlines, detailed discussions were held between researchers and initial testing was conducted with two family caregivers. Interviews were transcribed by researchers and check against audio recordings. Codes and themes were developed separately by two researchers and then compared and modified. Themes and transcripts were reviewed and compared by another neutral person.

FCTI is a valid and reliable scale with a Cronbach's alpha of 0.844, which was developed by Clark and Rakowski. ZBI scale was developed by Zarit et al. with a Cronbach's alpha of 0.733. Both two scales have good structural, content and face validity.

## Results

In the quantitative study, 76 family caregivers consisted 39 (51.3%) males and 37 (48.7%) females, with range of 21–60 years. 66 of children were female (86.8%) and 10 (13.2%) were male. Details can be seen in Table 1. In the qualitative interviews, 7 participants were females and 5 were males, aged between 21 and 54 years (Table 2).

**Table 1:** General information on caregivers and their children with DDH (*N* = 76).

Items		Caregivers	Children
Gender	Male	39 (51.3%)	10 (13.2%)
	Female	37 (48.7%)	66 (86.8%)
Age		35.1 ± 9.1	4.2 ± 2.8
Educational level	Elementary school	42 (55.3%)	
	Junior high school	26 (34.2%)	
	High school	5 (6.6%)	
	Junior college	2 (2.6%)	
	College	1 (1.3%)	
Monthly household income	Less than ¥3,000	51 (67.1%)	
	¥3,000–5,000	18 (23.7%)	
	¥5,000–8,000	4 (5.3%)	
	¥8,000–10,000	3 (3.9%)	
Relationship with child	Parents	61 (80.3%)	
	Grandparents	9 (11.8%)	
	Brother/Sister	1 (1.3%)	
	Other relatives (e.g., uncle)	5 (6.6%)	
Hip type	Subluxation		44 (57.9%)
	Dislocation		32 (42.1%)
Hip site	Left		11 (14.5%)
	Right		16 (21.1%)
	Bilateral		49 (64.5%)

**Table 2:** Information about interviewees and affected children (*N* = 12).

Participants	Gender	Age	Relationship	Children				
				Gender	Age	Hip site	Hip type	Treatment
P1	Female	36	Mother	Female	3	Left	Dislocation	Open reduction + Pelvic + Femoral osteotomy
P2	Male	26	Father	Female	2	Left	Dislocation	Open reduction + Pelvic + Femoral osteotomy
P3	Female	36	Mother	Female	3	Bilateral	Dislocation	Open reduction + Pelvic + Femoral osteotomy
P4	Female	37	Mother	Female	3	Bilateral	Dislocation	Open reduction + Pelvic + Femoral osteotomy
P5	Female	21	Mother	Female	2	Bilateral	Dislocation	Open reduction + Pelvic + Femoral osteotomy
P6	Female	27	Mother	Female	4	Bilateral	Dislocation	Open reduction + Pelvic + Femoral osteotomy
P7	Female	27	Mother	Female	2	Bilateral	Dislocation	Open reduction + Pelvic + Femoral osteotomy
P8	Male	52	Grandpa	Male	3	Right	Dislocation	Open reduction + Pelvic + Femoral osteotomy
P9	Male	54	Grandpa	Female	2	Bilateral	Dislocation	Open reduction + Pelvic + Femoral osteotomy
P10	Male	27	Father	Female	3	Bilateral	Dislocation	Open reduction + Pelvic + Femoral osteotomy
P11	Male	27	Father	Female	2	Right	Dislocation	Open reduction + Pelvic + Femoral osteotomy
P12	Female	45	Mother	Female	12	Bilateral	Dislocation	Open reduction + Pelvic + Femoral osteotomy

### Quantitative findings

Total score of FCTI and SSRS was (13.04 ± 7.16) and (45.13 ± 6.86), separately. Total score of ZBI was (29.87 ± 11.25), with role burden dimension score was (6.57 ± 3.30) and personal burden dimension score was (17.48 ± 5.88). When analyzing FCTI scores of each entry, we found that 55 (72.4%) chose “difficulty/extreme difficulty” for entry 5, “Give appropriate consideration to care-receiver's options and preferences”. Entry 7, “Supervise prescribed treatments and general recommendations” was selected as “no difficulty” by 61 person (80.3%). In analyzing scores for each entry of ZBI, it was found entry 7, “Are you worried about patient's future, in any way?” had the highest score, with majority of caregivers (81.6%) choosing “sometimes/often/always”. Entry 10, “Do you feel your health has suffered as a result of caring for the patient” was the lowest scoring, with 48 (63.1%) choosing “no” impact (Tables 3, 4).

**Table 3:** Descriptive analysis of family caregiver task inventory (FCTI).

Items	Median	IQR
1. Monitor course of condition and evaluate significance of changes	1	(0,1)
2. Normalise care-receiver routine, within bounds of the impairment	1	(0,1)
3. Perform basic ADL for the care-receiver	0	(0,1)
4. Gain knowledge about the disease	1	(0,1)
5. Give appropriate consideration to care-receivers options and preferences	1	(0,1)
6. Be available when needed	0	(0,0)
7. Supervise prescribed treatments and general recommendations	0	(0,0)
8. Evaluate strength/resources of the care-receiver	1	(0,1)
9. Cope with upsetting behavior of the care-receiver	0	(0,1)
10. Resolve guilt over ‘negative feelings’ towards care-receiver	0	(0,0.75)
11. Compensate for emotional drain from constant responsibility	0	(0,1)
12. Find a locus of blame for the condition/disease	0	(0,1)
13. Separate feelings regarding condition from feelings toward the care-receiver	1	(0,1)
14. Resolve uncertainty about one's skills as a caregiver	0	(0,1)
15. Release tensions/feelings toward the care-receiver	0	(0,1)
16. Anticipate needs for future assistance	1	(0,1)
17. Designate other responsible caregiver(s)	0	(0,1)
18. Manage feelings toward other family members who do not regularly help	0	(0,1)
19. Maintain the family as effective decision-making group over a long period of time	0	(0,1)
20. Interact with medical, health and social service professionals	0	(0,1)
21. Satisfy needs for creativity/originality to offset tedious routine	1	(0,1)
22. Avoid severe drain on physical strength/health	0	(0,1)
23. Make up for or avoid loss/restrictions on future plans and perspectives	1	(0,1)
24. Readjust personal routine	0	(0,0)
25. Compensate for disruption of sleep	0	(0,0)

Score range: 0 = no difficulty; 1 = difficulty; 2 = extreme difficulty.

**Table 4:** Descriptive analysis of zarit caregiver burden interview (ZBI).

Items	Median	IQR
1. Do you think that patients in your care will ask you for too much care?	1	(0,2)
2. Do you think that you will run out of time due to caring for patients?	1	(0,2)
3. Do you find it stressful to juggle caring for patients with trying to do your home and work?	2	(1,3)
4. Do you feel that being embarrassed by patients’ behavior?	1	(0,2)
5. Do you find it annoying to have patients around you?	0	(0,1)
6. Do you believe that your patient has affected your relationships with your family and friends?	0	(0,1)
7. Are you concerned about patient's future?	3	(2,4)
8. Do you believe that patients are dependent on you?	2	(1,3)
9. Are you nervous when patients are around you?	1	(0,2)
10. Do you believe that your health has suffered as a result of caring for patients?	0	(0,1)
11. Do you believe that you do not have time for your own personal affairs due to caring for patients?	1	(0,2)
12. Do you feel that your socialising suffers as a result of caring for patients?	0	(0,1)
13. Have you ever given up on the idea of having a friend over due to a sick person being home?	0	(0,2)
14. Do you feel that patient looks only to you for care, as if you are the only person she/he can rely on?	1	(0,3)
15. Do you believe that, except for your expenses, you have no money left over for patient care?	0	(0,2)
16. Do you think that you are likely to spend more time caring for patients?	3	(1,4)
17. Do you think that living your life the way you want to since you started caring is no longer possible?	1	(0,2)
18. Do you wish that you could leave your patients in the care of someone else?	0	(0,1.75)
19. Do you have a situation where you don't know what to do with a patient?	1	(0,2)
20. You think more should be done for patients, don't you?	2	(1,3)
21. Do you think you could do a better job of caring for your patients?	2	(2,3)
22. How would you rate your burden of care in general?	2	(1,3)

Score range: 0 = no; 1 = occasional; 2 = sometimes; 3 = often; 4 = always.

Results of Pearson correlation analysis suggested that scores of FCTI were positively associated with scores of ZBI (*r* = 0.512, *P* < 0.01) and negatively correlated with scores of SSRS (*r* = −0.309, *P* < 0.01). However, higher scores of FCTI indicated poor caring capacity. This implied that the poorer caregiving ability of family caregivers, the more severe caregiving burden. Moreover, the higher level of social support, the poorer caregiving ability (Table 5).

**Table 5:** Correlation analysis of study variables.

Variables	1	2	3
1. FCTI	–		
2. ZBI	0.512**	–	
3. SSRS	−0.309**	−0.166	–

** *P* < 0.01.

### Qualitative findings

#### Different emotions at different stages

Based on the analysis of qualitative data the following two themes emerged.

##### Initial worry

Many family members showed obvious anxiety and concern when they first learned of their child's illness, wondering if doctor had misdiagnosed. Others were puzzled and curious as to the cause of disease. For example, two family caregivers mentioned:

P4(female, age 37): I thought it was impossible when doctor told me that she had some problems with her hip joint, she’s been fine since birth. None of the other children in our family have had this situation, so I don’t understand. I was always anxious.

P9(male, age 54): My child had some problems with his walking posture, but I didn’t pay attention to it. The test result was hip dislocation, what was it? I’ve never heard of it before. What caused it? I was very worried!

##### Backend trust

Most of the interviewees were parents of children, who were receptive and their mood changed significantly after the diagnosis from initial worry and doubt to trust and support. For example, three family caregivers said:

P6(female, age 27): At first, our family members were very anxious when we knew she was sick. Then we heard from doctors in Shanghai that the treatment technology for this disease was very mature, so we trust them. Doctors in Shanghai are very good!

P10(male, age 27): This was the second time to Shanghai, the first time (surgery) we did the left side and this time we would do the right side. The doctor in Shanghai put me at ease (thumbs up).

P11(male, age 27): The medical conditions in our hometown were limited. Last year, Danzeng’s daughter also came to Shanghai for surgery. After surgery, her walking posture improved a lot, so I brought my daughter here this year…… No matter what kind of examination and treatment, I would cooperate, and I just hoped that my child could walk like a normal child in the future.

#### Caregiving process overwhelmed

Based on the analysis of qualitative data the following two themes emerged.

##### Role conflict

Interviewees in this study were adults, mostly parents of affected children, who, in addition to the responsibility of caring, also had social roles, such as those in workplace. For example, two family caregivers mentioned:

P2(male, age 26): I have already resigned from my job when I took my child to Shanghai for medical treatment. There were two elderly people in my family who were in poor health, and I was the only one who took care of them.

P5(female, age 21): I didn’t expect this to happen to my child. It’s a big expense, and my job wasn’t going well.

##### Lack of caregiving capacity

Developmental hip dislocation is a special disease, and caregiving involves plaster care, dietary guidance, rehabilitation exercises, life care, and other aspects. Caregivers need to have a wealth relevant knowledge and caregiving skills to cope with various emergencies that arise in children. The educational level of interviewees in this study was generally low, and 10(83.3%) of family members talked about how they felt overwhelmed by the caregiving process and lacked energy to take care of their children because of their own lack of ability. For example, two family caregivers said:

P1(female, age 26): The most troublesome part of caring for a child was changing her nappy. Because she was been wearing a cast since her surgery, my hands were full every time when I changed her nappy.

P8(male, age 52): I am his grandfather and had never attended to school. Nurse told me how to exercise his lower limbs, but I couldn’t understand. I dared not move his legs casually, so he had hardly exercised.

#### Insufficient information

Based on the analysis of qualitative data the following two themes emerged.

##### Disease-specific information

Caregivers had limited access to disease knowledge, there was a lack of evidence about the source and authenticity of information which they obtained from Internet. Moreover, the complexity of information on the Internet could easily mislead families, even delayed treatment of child. In addition, due to regional language differences, some family members need to go through an interpreter to communicate with healthcare workers, and there may be some communication errors. For example, two family caregivers said:

P3(female, age 36): A year ago, my child walked like a duckling, and the examination results indicated a hip joint issue. We learned from Baidu that this condition didn’t require surgery, so our family didn’t take it seriously. However, this year, when doctors in Shanghai re-examined, they informed us that surgery was necessary. Consequently, we traveled to Shanghai with the medical team.

P12(female, age 45): I can’t speak Mandarin, so I didn’t understand what doctors and nurses were saying. Although there was a translator, it was very inconvenient for us to communicate. It would be great if there were a specialized introduction in Tibetan about disease.

##### Actual care information

Many family caregivers mentioned that they had difficulties in caring for postoperative children and were eager to obtain more information about home care from medical staff, such as daily life care, plaster care, psychological care, and other aspects. For example, three family caregivers mentioned:

P6(female, age 27): At hospital, there were many nurses to assist us. But what should we do when we go home? Whenever I tried to turn her over, she cried out in pain, which made me hesitate to move her……The weather was hot now, and since she was wearing a cast, it was not easy for me to give her a bath. Today, she even wet the cast during urination. I am not sure if this will have any adverse effects. There were just so many issues.

P7(female, age 27): After the surgery, she kept crying. Today, she even got angry with me, complaining of leg pain and discomfort, and refused to eat. Oh, I was exhausted by her.

P11(male, age 27): This was the only child in our family, we were usually quite precious. It was very hard for me to see her in pain. I wanted to hold her but I was afraid to do that because of the cast, so we parents were at s loss as to what to do. I wish doctors and nurses could tell us what to do, but you were usually very busy and I was too embarrassed to ask some questions.

### Social support

Economic status is an important influence on the burden of care for caregivers of children with DDH. However, Shanghai Children's Hospital and People's Hospital of Shigatse, Tibet, jointly launched a public assistance program, “Love of Gesang Flower”, in 2014 to support some of children with DDH for surgical treatment. The program alleviated economic pressure on some families and contributed to logistical support work for caregivers. Eight (66.7%) of caregivers interviewed expressed their recognition of the program. For example, two family caregivers mentioned:

P4(female, age 37): This program was fantastic! My family’s financial situation wasn’t good, and initially, I was deeply concerned about the high cost of surgery. Now, with the support of this project, I did not have to worry about money. I was deeply grateful to Shanghai Children’s Hospital.

P5(female, age 21): Shanghai boasted excellent policies, skilled doctors, and a good service attitude, making it an excellent place in every aspect.

### Mixed methods findings

Themes derived from qualitative findings were compared and merged with quantitative findings, including level of caregiving competence of family caregivers, perceptions of caregiving process, and burdens incurred while caring. The findings were confirmed and extended by comparing and merging the data.

Five themes: initial worry, backend trust, role conflict, insufficient disease-specific information and social support confirmed the quantitative findings. The themes of lack of caregiving capacity and insufficient actual care information provided an expansion of the quantitative findings. In the survey, caregivers of children with DDH in Tibet showed a low level of caregiving capacity with scores of (13.04 ± 7.16) and moderate level of caregiving burden with scores of (29.87 ± 11.25). Due to long cycle of care for DDH, a greater burden of care was more likely to arise when there was insufficient capacity for caregivers. Additionally, in response to lack of information about actual care, caregivers indicated that casts were a major deterrent to their ability to perform a range of daily care tasks. But since casts needed to be worn for 2–3 months, this posed a great challenge in caring (Table 6).

**Table 6:** Comparison of caregivers’ experiences of children with DDH.

Themes		Quantitative	Qualitative quotes	Inferences
Different emotions at different stages	1. Initial worry	1. Caregivers indicated that they sometimes felt stressed when caring for children. [Median = 2, IQR = (1,3)] 2. Caregivers indicated that they often worried about children's future. [Median = 3, IQR = (2,4)]	*“I thought it was impossible when doctor told me that she had some problems with her hip joint……I was anxious all the time.” (female, age 37)* *“My family was very worried about whether he would be able to walk properly.” (male, age 52)*	**Confirmation** Family caregivers were often tense and anxious in the early stages of leaning about their child's illness and showed significant negative emotions.
	2. Backend trust	41 Caregivers (53.9%) indicated that they could better release tensions when facing patient.	*“The doctor in Shanghai put me at ease.” (male, age 27)* *“At first, we were very nervous. Then we heard that the treatment was very mature, so we trust them.” (male, age 27)*	**Confirmation** Family caregivers transformed from worried and nervous at the initial diagnosis to trusting and supportive after surgery, demonstrating different moods at different stages of treatment.
Caregiving process overwhelmed	1. Role conflict	Caregivers indicated that they sometimes had difficulty between household chores, work and childcare. [Median = 2, IQR = (1,3)]	*“I had resigned from my job when I took child to Shanghai for treatment because of the elderly’s poor health, no one but me could take care of her.” (female, age 26)* *“My family was not very well off, and I not only had to work, but also take care of my child.” (female, age 45)*	**Confirmation** Family caregivers tended to play different roles, including roles of parent, employee, and educator. When child was ill, they took on the task of caregiving, other roles such as staff may be in conflict.
	2. Lack of caregiving capacity	Caregivers indicated that they often spend more time caring for children. [Median = 3, IQR = (1,4)] 2. The total score of FCTI was (13.04 ± 7.16).	*“I was his grandfather and had never attended to school. I moved slower in my old age and it was a bit of a struggle to take care of child.” (male, age 52)* *“After operation, she cried all the time because of the pain in her leg. I saw her crying and felt very bad, I didn’t know how to ease this bad mood.” (female, age 27)*	**Expansion** Family caregivers were at a low level of caregiving competence. In addition to parents, grandparents were often involved in caregiving process and even dominated it.
Insufficient information	1. Disease-specific information	1. Caregivers indicated that they had difficulty in observing children's condition and assessing changes in condition. [Median = 1, IQR = (0,1)] 2. Caregivers indicated that they had difficulty in increasing disease-related knowledge. [Median = 1, IQR = (0,1)]	*“We didn’t know anything about the disease, let alone that it required surgery. We would love to learn more about information of disease, but it was clear that we were lacking in competence.” (female, age 21)* *“My child had a major osteotomy and her vital signs needed to be monitored after surgery. But I couldn’t understand the data on cardiac monitor and detect any changes in her condition in time.” (female, age 27)*	**Confirmation** Family caregivers generally felt they lacked of disease-specific information prevented them from accurately assessing their child's condition in a timely manner.
	2. Actual care information	57 Caregivers (75.0%) indicated that they had a moderate or higher caregiving burden.	*“Although nurse had told us she was okay to eat something. But I didn’t know which kind of food she could eat and couldn’t eat.” (male, age 27)* *“We were worried about complications after surgery.” (female, age 21)* *“I couldn’t understand how to exercise her lower limbs.” (male, age 52)* *“After surgery, she had been in bed with a cast on her lower leg. I didn’t know how to bathe her.” (female, age 27)*	**Expansion** Family caregivers had many problems with caregiving, mainly including plaster care, continence care, skin care, and rehabilitation exercises.
Social support		1. The total score of SSRS was (45.13 ± 6.86), subjective support dimension was (27.69 ± 4.14), objective support dimension was (9.79 ± 3.09), and utilization of social support dimension was (7.64 ± 2.13).	*“The ‘Gerbera Love Program’ helped us alleviate financial stress.” (female, age 37)* *“Shanghai’s healthcare policies had been very supportive and helpful, thanks to them!” (female, age 52)*	**Confirmation** Family caregivers were supported by the public welfare assistance program during their hospitalization, which greatly reduced medical burden of children in Tibetan.

## Discussion

Shanghai Children's Hospital creates a comprehensive treatment and living environment for caregivers. Firstly, medical staff placed them in the same ward, making it easier for caregivers to communicate with each other about illness and points of caring. For caregivers who cannot communicate in Mandarin, this arrangement also facilitates centralized translation by interpreters and improves work efficiency. Secondly, hospital provides caregivers with basic hospitalization necessities such as beds, quilts, meals, and daily necessities, which greatly reduces burden of caregivers' life, so that they do not have to worry about basic life and can concentrate on receiving treatment. Thirdly, hospital also arranges special cars and nursing staff to pick up and drop off children and their caregivers at designated train station to ensure their safety as much as possible.

This mixed-method study utilized quantitative and qualitative methods to a comprehensive understanding of caring burden and experiences of family caregivers of children with DDH in Tibet, China. This provided greater insight than that gained by using either type of data in isolation, as quantitative method allowed for a greater degree of statistical generalization, while qualitative method provided deeper insight and a more nuanced view of phenomenon (23). Quantitative analysis of standardized questionnaires was used to determine status of caregiving burden and caregiving capacity of family caregivers of children with DDH, and qualitative analysis of semi-structured interviews was used to better understanding of caregiving experiences. Mixed-methods analyses were conducted to assess degree of agreement and disagreement between quantitative and qualitative results.

Caregiver capacity is ability of caregiver to effectively meet physical, emotional, and social needs of patients while maintaining their own health (28). This capacity includes knowledge, skills, resources and support systems available to caregivers. Findings from this study of FCTI showed that caregiving capacity was at a low level. Entries 1, 2, 5, 8 and 13 were the most difficult things for family caregivers to do with children. Of these, entries 1, 2, 5 belong to “Learning to cope with new role” dimension, entry 8 to “Providing care according to care-receiver's needs” dimension, and entry 13 to “Managing own emotional needs” dimension. These were in convergence with the findings of in-depth interviews. They reported not being able to adjust one's personal roles well enough to move from a work role to a caregiver role. In 1995, a scholar first introduced the concept of family caregiver role acquisition, which was a specific type of role transition occurring in families in response to changes in health (29). Some researchers showed family caregivers had low level of caregiving preparedness at discharge, which led to high caregiving burden in later weeks following discharge (30, 31). Adverse emotions such as distress and depression occurred during role transition (31). These results were congruent with some studies in the literature (20, 22, 32).

Caregiver burden is defined as the distressed results from providing care (33). Suffering from a reduction in free time and a lack of rest because of the influence of surgery tasks, caregivers are likely to feel burdened. Family caregivers’ burden was at a moderate level in this research. Entries 3, 7, 8, 16, 20, 21 were the ones that families found most burdensome to care for. Qualitative interview themes expanded on the findings of this part of the quantitative study, with caregivers indicating that burden was primarily caused by a lack of caregiving capacity. Due to lack of knowledge about disease, wearing a cast after surgery hindered all aspects of daily life care, including continence care, skin cleaning, and exercise. The average time for postoperative children to wear plaster is 8 weeks, during which lower limb functional exercise is required. However, due to distance and language issues, the effectiveness of functional exercise cannot be effectively evaluated. The uptake in caregiver burden in post-discharge has significant implications for patient's outcomes, such as symptom management, recovery, hospital re-admission, and caregivers’ health and well-being (30). It had been suggested that a structured education and training program for caregivers improved pre-discharge preparation, reduced caregiver's burden, and led to better outcomes for children (34). Extensive evidence suggested that caregiving burden was associated with mental and physical health problems (35–37). Strategies such as peer mentoring and peer support groups can also improve preparation, mental health and ability to handle stress of caregiving (38).

In addition, correlation analyses in quantitative study indicated that caregiving capacity was positively associated with caregiving burden and negatively correlated with social support. Some researchers suggested that social support mediated the relationship between caregiving capacity and caregiving burden (39). Previous researchers have demonstrated that social support is strongly associated with caregiver burden (40). Caregiving can be a lonely and demanding role, and social support is vital to mitigate the negative effects of caregiver burden (41). When caregivers felt socially supported-whether through family, friends, or community resources-they were more likely to develop resilience to cope with challenges of caregiving (42). Moreover, enhancing level of social support through some interventions (support groups or community resources) can significantly improve overall quality of life of caregivers. By meeting emotional and practical needs of caregivers, their burden can be reduced and their ability to care increased (43). Thus, it followed that role of strengthening the level of social support is crucial in development of family caregiving strategies. The children in this study benefited from the “Love of Gesang Flower” public welfare program, which paid for all costs of children's families, including surgical fees, transportation, and living expenses, which greatly reduced financial burden on their family. In addition, the program trained and taught community healthcare workers in Shigatse through courses on “Early screening, diagnosis and treatment of DDH”, teaching workshops, and on-site instruction in hip ultrasound and surgical skills. These initiatives help residents better understand the causes, clinical manifestations and treatment of DDH.

This study is part of the construction of a home care program for children with DDH in Tibet. Research was targeted and directed to caregivers of children with DDH in the highlands, and presented caregiving ability and burden levels using a convergent mixed-methods research, which enriched findings in the field of DDH nursing. Results were analyzed from caregivers’ perspective and study methodology was reproducible, which were useful and informative in other countries and regions. Comparing quantitative and qualitative data can improve our understanding of caring experiences of family caregivers in Tibet, China. Based on these findings, it is recommended that requirements for postoperative home care for children with DDH in Tibet deserves attention and measures should be taken to alleviate caregivers’ burden. Healthcare professionals should develop a targeted home care management program so that families can fully understand various care measures after discharge, including daily care, plaster care, skin care, continence care and so on. Internationally, more research is needed to identify strategies for reducing caring burden against family caregivers of children with DDH. With continuous development and improvement of AI technology, intelligent remote mutual medical model will be more conducive to the communication and exchange between healthcare professionals and children at a distance. Therefore, future research may need to be conducted in conjunction with AI to reflect the characteristics of the times.

## Limitations

Purposive sampling was used for quantitative portion of this study, a method that may limit generalization to other similar populations. Insufficient sample size and single source of subjects affected credibility of study. In addition, as a non-intervention study, we were unable to recommend causality of the phenomenon from findings. Therefore, sample size should be increased and multicenter studies should be conducted in the future.

## Data Availability

The raw data supporting the conclusions of this article will be made available by the authors, without undue reservation.
